# Hybrid Internal Combustion Engine Based Auxiliary Power Unit

**DOI:** 10.3390/mi11040438

**Published:** 2020-04-21

**Authors:** Vladimir Yuhimenko, Dmitry Baimel, Moshe Sitbon, Moshe Averbukh, Simon Lineykin, Alon Kuperman

**Affiliations:** 1 School of Electrical and Computer Engineering, Ben-Gurion University of the Negev, Beer-Sheva 8410501, Israel; yuhimenko.vladimir@gmail.com; 2 Department of Electrical and Electronics Engineering, Shamoon College of Engineering, Beer-Sheva 84100, Israel; dmitrba@sce.ac.il; 3 Department of Electrical Engineering and Electronics, Ariel University of Samaria, Ariel 40700, Israel; moshesi@ariel.ac.il (M.S.); mosheav@ariel.ac.il (M.A.); simonl@ariel.ac.il (S.L.)

**Keywords:** auxiliary power unit, internal combustion engine, energy management, supercapacitors, specific fuel consumption

## Abstract

The brief presents some principles of the ON/OFF operational strategy applied to energy management of a hybrid internal combustion engine (ICE) based auxiliary power unit (APU). It is shown that significant reduction of fuel consumption (78% for the example system presented) and maintenance expenses (80% operation time decrease was attained by the system) may be achieved by such a strategy, shifting the system operation point towards corresponding optimal region. The side effect of aggravated amount of starting events is cured by employing an actively balanced supercapacitor (SC)-based emergency starter (SCS). The SCS operates as short-time energy storage device, charging from the battery at a low rate and then providing a current burst required for proper internal combustion engine starting. Current sensorless method of automatic connection (based on bus voltage sensing) and disconnection (based on sensing the voltage across bidirectional MOSFET-based switch) of the SCS is also proposed. The proposed circuitry, successfully validated by experiments, may be arbitrarily scaled up or down according to application rating.

## 1. Introduction

**Figure 1:**
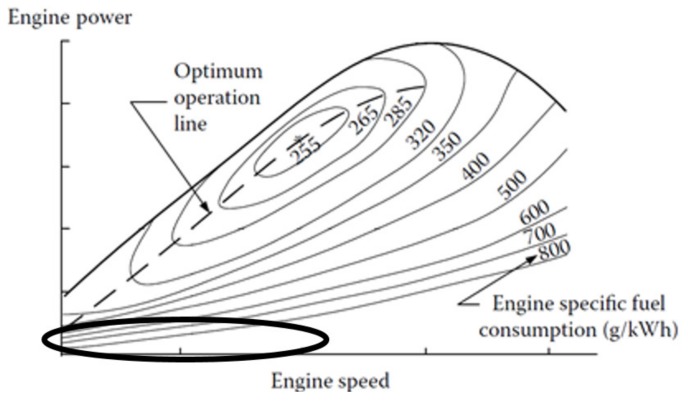
Specific fuel consumption map of a typical internal combustion engine.

Armored combat vehicles and heavy trucks are among major transportation fuel consumers, equipped with horsepower-rich (typically diesel) internal combustion engines (ICEs) rated up to several megawatts. While propulsion loads are probably the major energy consumers, substantial amount of electrical loads is also present onboard. Electrical system of such vehicles is typically based on a 28 V DC bus, fed by an alternator connected to the driving shaft and buffered by lead-acid batteries. When the main ICE is on, it feeds both propulsion and auxiliary electrical loads while replenishing batteries via an alternator. On the other hand, upon main ICE inactivity, electrical loads are energized by the battery pack. In case the main ICE is OFF for long periods of time, yet significant electrical load is present, the battery pack is quickly depleted, calling for ICE start followed by nearly-idle operation to supply the required electrical power [1]. Light load ICE operation is extremely inefficient [2,3,4], as shown in Figure 1. Moreover, maintenance period of an ICE engine depends on operating duration rather than on output power. Hence, continuous nearly idle operation leads to increased fuel consumption and aggravated maintenance requirements [5,6].

**Figure 2:**
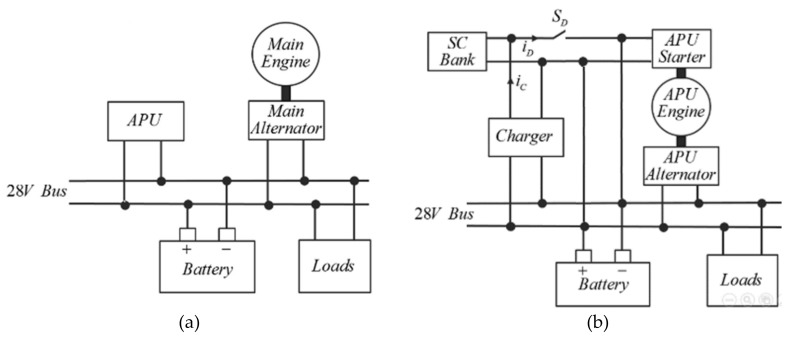
Block diagram representation of auxiliary power unit (APU)-assisted heavy vehicle electrical system. (**a**) General arrangement and (**b**) with a supercapacitor-based emergency starter (SCS) starting device.

As a solution to these shortcomings, small auxiliary power units (APUs), shown in Figure 2a, are often installed in heavy vehicles, where frequent mechanical propulsion inactivity under significant auxiliary electrical loads is expected. Such APUs automatically come into operation when the main ICE is OFF. Unfortunately, APUs are usually rated according to the maximum expected auxiliary load (as opposed to near-average load value in non-critical applications [7]), while the probability of rated load appearance is extremely low, i.e., most of the time, APU supplies float batteries charge and background loads, operating well-below its rated power. As a result, light load operation issues of the main ICE are transferred to the APU.

Several optimal [8] and adaptive [9] energy management strategies were proposed and examined in the literature. System performance under these fairly complicated control structures is highly dependent on correct system representation and available information, which are not always available in real-time operating conditions. On the other hand, ON/OFF operational strategy [10] does not require detailed system information, and while not being optimal, proposes excellent trade-off between simplicity and performance. In this brief, ON/OFF APU operation is examined instead of the continuous one, significantly reducing fuel consumption while prolonging the period between successive maintenances.

A major drawback of the ON–OFF operational strategy is the increased amount of APU engine starting events [3], imposing multiple undesired stress on the vehicle battery. Engine starting procedure requires an electrical source, capable of providing short yet intensive current burst. One of the promising solutions to starting issues was recently revealed in [11], where a supercapacitor (SC) bank was utilized as an intermediate energy storage, capable of charging from a battery (or any other external source) at low rate *i_C_* (cf. Figure 2b) via a dedicated unidirectional charger (typically of buck-boost topology). Once fully charged, the SC bank provides the required current burst *i_D_* to the APU starting motor via the switch *S_D_*. Upon SC-base starting, some current is drawn by the batteries as well; nevertheless their impedance is much higher than that of the starting motor, hence the latter would pull the majority of the current from the SC. Upon starting process completion, APU starting motor disconnects and APU alternator begins providing current to the DC bus. Since the SC bank presents a much lower impedance than does the battery, it may draw the majority of alternator-provided current in case *S_D_* is bidirectional. This may cause two issues. First, low SC impedance [12,13] may cause alternator overcurrent and subsequent protection circuitry tripping. Alternatively, alternator-provided current may exceed rated continuous SC current and damage the device. Thus, the SCS must be disconnected from the DC bus upon alternator connection.

To summarize, a dedicated controller must properly detect both starting process initiation and completion instants. In this brief, a solution based on voltage sensing (eliminating the need for high starting current sensing) is proposed and validated. Experimental verification of the proposed ON–OFF operation of ICE-based APU with SC-based emergency starter (SCS) reveals the method feasibility.

## 2. Operational Strategy

**Figure 3:**
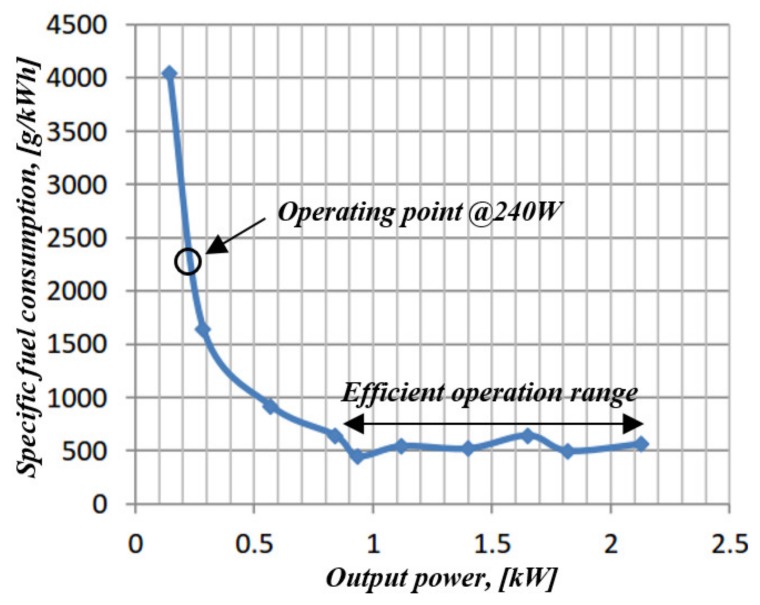
Measured APU specific fuel consumption versus output power.

As a case study, consider a 28 V DC APU (typical for small armored vehicles), consisting of a 3.4 kW Yanmar L48AE diesel ICE propelling a 2 kW Balmar MEP-501A alternator, terminated by a three-phase diode bridge [14]. Figure 3 presents a plot of measured full-throttle specific fuel consumption of the APU versus output DC-side electrical power. 

It is well-evident that specific fuel consumption remains relatively constant within a 0.9 kW output power range but increased sharply when the output power was further reduced. When operated against a 60 Ah Volta lead-acid battery bank as a typical load, the APU supplies most of the time circa 0.24 kW of the DC power (floating battery charging [15,16]). According to Figure 3, specific fuel consumption of the APU @0.24 kW load was 4.7 times higher than the @>0.9 kW load range. It is hence proposed to operate the APU in the ON/OFF fashion, bringing it into operation mode either when the batteries state of charge becomes low or upon high-demanding load detection. When charging power becomes low, i.e., either the batteries are replenished or high-rating load is disconnected, the APU is shut down. Since optimal detection of ON/OFF instants requires full exact knowledge about the system [3,10], a simple solution is proposed next.

**Figure 4:**
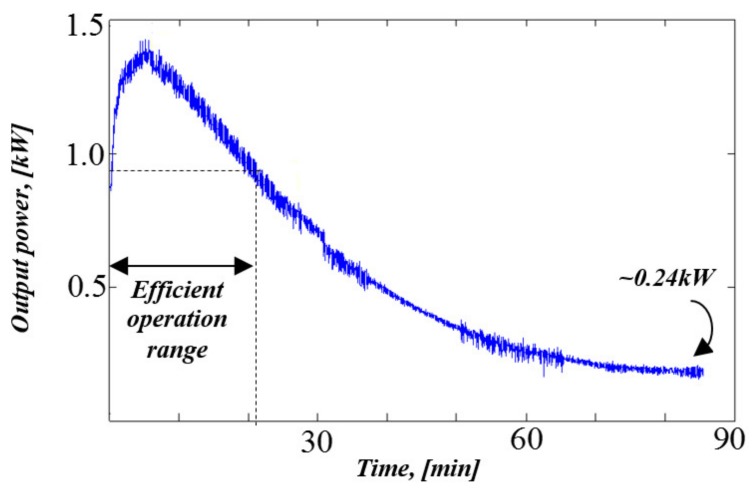
Measured APU output power versus time during depleted lead-acid battery charging.

**Figure 5:**
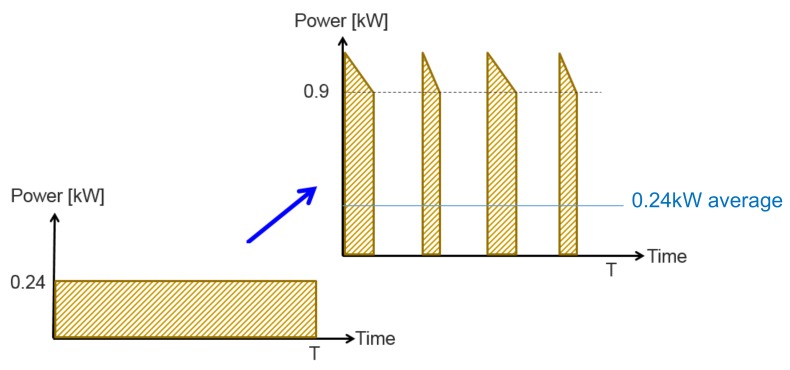
Continuous to ON/OFF operational principle transition.

Typical measured output power of the APU versus time behavior when connected to a low state-of-charge 60 Ah lead-acid battery is shown in Figure 4. Since the APU operated as a voltage source, charging power was imposed by the battery pack. First, APU power gradually rose due to reduction of the battery internal resistance. Then, charging power monotonically reduced according to state-of-charge rise. It might be concluded that during the first 21 minutes, the APU remained within efficient operation range and then entered the inefficient zone. Eventually, APU output power settled around 0.24 kW continuous operation load. Consequently, it was proposed to shut the APU down when its output power reduced below 0.9 kW, as shown schematically in Figure 5.

It should be emphasized that the energy delivered by the APU in both cases was nearly the same (increased transmission losses—negligible compared to fuel savings—are expected when operating with higher output power due to higher root mean square (RMS) value of charging current); nevertheless, the operation duration and fuel consumption were cordially different, as shown in the Verification Section below. 

However, the proposed operating principle suffered from two main drawbacks. First, lead-acid batteries are prone to memory effect when not fully charged repeatedly. Shutting the APU down at 0.9 kW did not allow charging the battery to a 100% state of charge. Nevertheless, recall that APU-based operation is required only upon main engine inactivity. During normal operation, the main ICE is ON and the batteries were fully recharged as required. Therefore, capacity loss due to occasional incomplete charging was expected to be insignificant. Second, ON/OFF operation requires frequent APU engine starting, imposing additional stress on the battery, which shortens its life. To avoid this, it was proposed to pass the starting stress from the battery to an SCS [11], which allows increasing battery service life and adds flexibility to the system. The proposed system is shown in Figure 2b. When the starting event was approaching, the charger began filling the SC bank from the main DC bus at a predetermined rate. When the SC bank was full, the charger was deactivated and switch *S_D_* was closed, connecting the SC based starter to the APU starting motor and the 28 V bus. The starting event was detected upon low battery state-of-charge or high-rated load connection, as explained in the subsequent section.

## 3. SCS Connection/Disconnection Timing

Denoting battery open circuit voltage and internal resistance as *v_B_* and *r_B_*, respectively, its terminal voltage *v_O_* upon non-SCS-assisted starting is given by

(1) $$v_{O}=v_{B}-r_{B}i_{B}$$

with *i_B_* denoting the starting current. As explained in [14], in order to start an ICE, the cranking motor must be accelerated to a minimum predetermined speed, which is proportional to the minimum battery terminal voltage *v_O,_*_min_. Therefore, once due to discharge/temperature/ageing battery internal resistance increases beyond

(2) $$r_{B,\max}=\frac{v_{B}-v_{O,\min}}{i_{B}},$$

**Figure 6:**
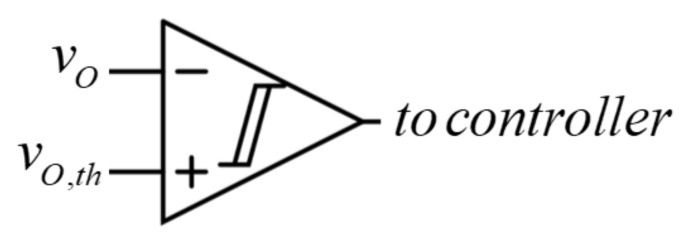
SCS connection timing circuit.

The starting process would be unsuccessful, i.e., battery terminal voltage would drop below *v_O,_*_min_. It should be emphasized that the vehicle computer often sets a minimum allowed battery voltage to a value higher than *v_O,_*_min_ for the protection purpose (e.g., 12 V in vehicles with a 24 V DC bus). Therefore, by monitoring the voltage at SCS terminals it is possible to recognize a starting event by detecting an abrupt voltage drop below a preset threshold value *v_O,_*_th_, as shown in Figure 6.

**Figure 7:**
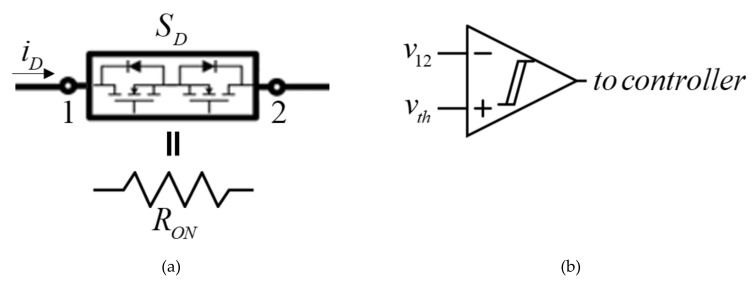
Utilizing MOSFET ON-resistance as a current-sensing shunt. (**a**) Switch *S_D_* structure; (**b**) SCS disconnection timing circuit.

It is well-known that a starter current possesses a triangular-like shape [17], i.e., following a sharp peak, the current gradually reduces. When starting is completed, the cranking motor is disconnected (i.e., starting current drops to zero) and then alternator excitation field is activated. Consequently, the best way to detect starting process termination is monitoring the current and detecting zero crossing. Unfortunately, this requires adding a bulky and expensive current sensor to the system. It is interesting to note that the starting process is actually over slightly after the starting current possesses the sharp peak, i.e., even though the cranking motor keeps drawing some amount of current, it does not influence the process anymore. This means that if the SCS is disconnected from the cranking motor before the current goes to zero, this would have no effect on the system. Consequently, it is proposed to utilize the ON-resistance *R_ON_* of the switch *S_D_* in Figure 2b (typically realized by two series connected MOSFETs, as shown in Figure 7) as shunt resistance for measuring the voltage drop *v_12_* across the switch, comparing it to a preset threshold value *v_th_* and getting the information regarding starting event termination to the controller.

Even though actual ON-resistance of the switch may differ from the one adopted from the datasheet, resulting current measurement error would not affect the detection since the range of currents suitable for SCS disconnection is wide.

## 4. Verification

**Figure 8:**
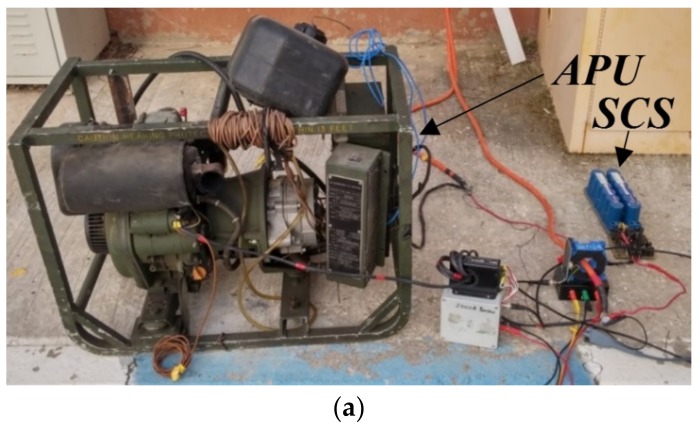

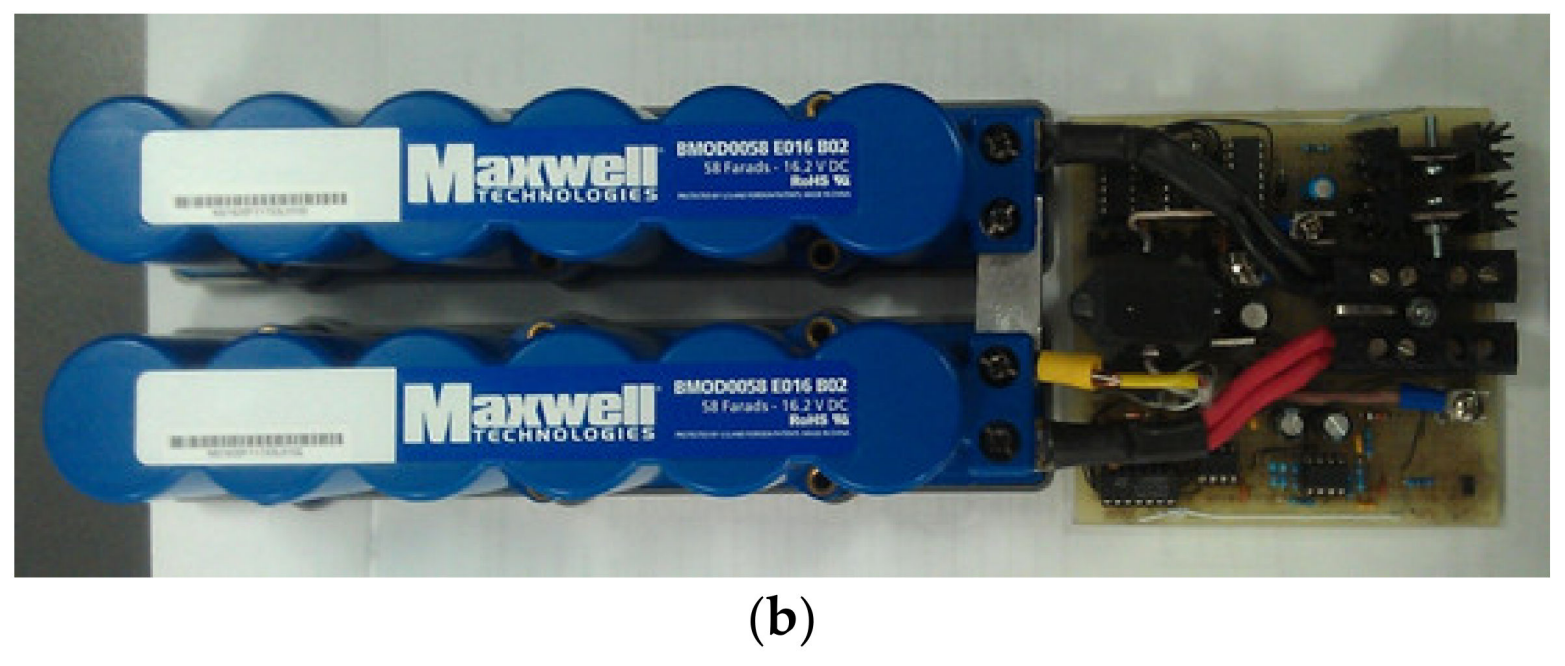
Experimental setup. (**a**) Overall view. (**b**) The SCS.

During experiments, the NHR-4700 electronic load, operating in a constant power mode, was used as the system load. A 29F SC bank was realized by a series connection of two Maxwell BMOD0058-E016-B02 58F supercapacitors [18,19] with an active balancer [20], required to avoid overcurrent in case of supercapacitors misbalance [21]. The supercapacitor charger was realized by a unidirectional 10 A non-inverting buck-boost converter [22]. For fuel consumption measurement, the fuel tank was placed on electronic weighting machine, passing an instantaneous weight value to a PC. Output power was measured by a power meter, connected across the APU output. Experimental prototype is depicted in Figure 8. During the first experiment, the APU was operated in the classical way, as shown in Figure 2a. Once the charging power reached a steady-state, the fuel consumption calculation was initiated and lasted 6 hours. During the second experiment, the APU was operated in the proposed way, as shown in Figure 2b. Again, the system was brought to a steady-state and then the proposed energy management strategy was enabled and fuel consumption calculation was initiated. The system was run until the generated output energy was equal to the amount attained during the first experiment (1.44 kWh). Comparison between continuous and ON/OFF operation is summarized in Table 1. It may be concluded that transition from continuous to ON/OFF operation yielded 5-times operational time and 4.6 times fuel consumption reductions, respectively. Fuel reduction of less than 5 times was associated with an increase battery conversion losses, mentioned above.

**Figure 9:**
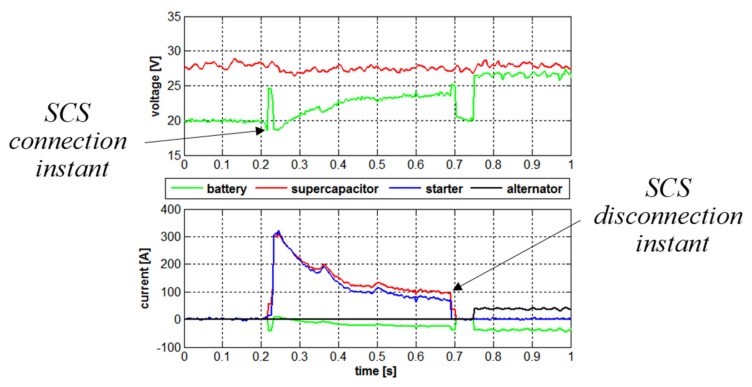
Experimental results—SCS connection and disconnection instants.

During the ON/OFF operation strategy experiment, the SC bank was charged to 27 V and lead-acid batteries were discharged down to 20 V. Starting detection under-voltage limit was set to 18 V in the SCS controller. At t = 0.2 s starting was initiated. As shown in Figure 9, the battery voltage immediately dropped down to 18 V. This was detected by an SCS controller and the SC was immediately connected to the 28 V bus by closing the bidirectional switch *S_D_* (cf. Figure 2b). Straightaway after this, the starting current began rising while some of it had flown into the battery since in the beginning of the process cranking motor presented high impedance as well (observe the momentary rise of battery voltage caused by the charging current). At t = 0.24 s, the starting current reached maximum value, provided by both SC and the battery (the former had provided most of it, of course). When the starting current began to descend, the SC provided the current to both cranking motor and the battery. Around t = 0.7 s, SCS controller detected a low starting current value and disconnected the SC from the DC bus. At t = 0.75 s, the alternator was connected to the 28 V bus and began charging the battery, as expected.

## 5. Conclusions

The brief revealed that ON/OFF operational strategy applied to ICE-based APU might significantly reduce fuel consumption and period between successive maintenances. For the given example system, the 80% operating period duration decrease and 78% fuel consumption reduction were attained for a typical operation cycle. On the other hand, it was shown that the above benefits came at the expense of an increased amount of starting events. In order to cope with the issue, a supercapacitor-based emergency starter was introduced to absorb the multiple starting stresses. A method of automatic connection and disconnection instants detection for the proposed add-on was proposed to synchronize the emergency starter with the rest of the system. The suggested circuitry is based on voltage sensing only, avoiding the use of current sensors. The suggested system functionality was successfully validated by experiments.

## Figures and Tables

**Table 1:** Experimental results of applying ON/OFF strategy versus continuous operation.

Strategy	Average Power When ON (kW)	Operating Time (h)	Output Energy (kWh)	Fuel Consumption (kg)
**Continuous**	0.24	6	1.44	3.32
**ON/OFF**	1.2	1.2	1.44	0.72
